# Virtual cooperativity in myoglobin oxygen saturation curve in skeletal muscle *in vivo*

**DOI:** 10.1186/1476-5918-5-3

**Published:** 2006-01-24

**Authors:** Akitoshi Seiyama

**Affiliations:** 1 Division of Physiology and Biosignaling, Osaka University Graduate School of Medicine, 2-2 Yamadaoka, Suita, Osaka 565-0871, Japan

## Abstract

**Background:**

Myoglobin (Mb) is the simplest monomeric hemoprotein and its physicochemical properties including reversible oxygen (O_2_)binding in aqueous solution are well known. Unexpectedly, however, its physiological role in intact muscle has not yet been established in spite of the fact that the role of the more complex tetrameric hemoprotein, hemoglobin (Hb), in red cells is well established. Here, I report my new findings on an overlooked property of skeletal Mb.

**Methods:**

I directly observed the oxygenation of Mb in perfused rat skeletal muscle under various states of tissue respiration. A computer-controlled rapid scanning spectrophotometer was used to measure the oxygenation of Mb in the transmission mode. The light beam was focused on the thigh (quadriceps) through a 5-mm-diameter light guide. The transmitted light was conducted to the spectrophotometer through another 5-mm-diameter light guide. Visible difference spectra in the range of 500–650 nm were recorded when O_2 _uptake in the hindlimb muscle reached a constant value after every stepwise change in the O_2 _concentration of the buffer.

**Results:**

The O_2 _dissociation curve (ODC) of Mb, when the effluent buffer O_2 _pressure was used as the abscissa, was of a sigmoid shape under normal and increased respiratory conditions whereas it was of rectangular hyperbolic shape under a suppressed respiratory condition. The dissociation curve was shifted toward the right and became more sigmoid with an increase in tissue respiration activity. These observations indicate that an increase in O_2 _demand in tissues makes the O_2 _saturation of Mb more sensitive to O_2 _pressure change in the capillaries and enhances the Mb-mediated O_2 _transfer from Hb to cytochrome oxidase (Cyt. aa_3_), especially under heavy O_2 _demands.

**Conclusion:**

The virtual cooperativity and O_2 _demand-dependent shifts of the ODC may provide a basis for explaining why Mb has been preserved as monomer during molecular evolution.

## Background

Mb is a monomeric hemoprotein with a molecular weight of 17 kDa, carrying a single oxygen (O_2_)-binding site per molecule. It is located near the contractile elements and cell membranes in the red skeletal and cardiac muscles of vertebrates [1]. Previously, Millikan [2,3] proposed the following three possible physiological functions for Mb: (a) an O_2 _store during temporary deficits in O_2 _supply, (b) an intracellular O_2 _transport agent and (c) an intracellular catalyst. Among them, the first function has traditionally been accepted. In the muscles of a beating heart and exercising skeletal muscles, Mb acts as a short-term O_2 _store (i.e., an O_2 _buffer), tiding the muscles over from one contraction to the next. The rich Mb content in skeletal muscles in aquatic mammals is considered to provide a long-term O_2 _store during diving. However, this role of Mb, at least in human, is not significant because its oxygen storage capacity is so low that the total oxygen bound to Mb is exhausted within ca. 5.5 s after being cut off from the O_2 _supply [4]. The second function, called "facilitated O_2_-diffusion by Mb", was based on findings in *in vitro *experiments [5,6]. The conditions required for this facilitated diffusion to occur are [7]: (a) existence of deoxygenated Mb in a certain fraction (or certain low intracellular partial pressure of O_2_), (b) existence of a spatial gradient of oxygenated Mb concentration as a driving force for translational diffusion of Mb, and (c) sufficient mobility of the oxygenated Mb to permit diffusion. Although this mechanism has been widely accepted, several discrepancies remain unresolved [8-12]. As for the third function, Doeller and Wittenberg [13] proposed the occurrence of Mb-mediated oxidative phosphorylation in heart myocytes under aerobic conditions. However, Mb concentration is not closely related to the oxidative capacity of muscles, that is, the concentration is higher in skeletal muscles (~0.5 mmole/kg wet wt.) than in heart muscles (~0.25 mmole/kg wet wt.) [7].

Thus, the physiological roles of Mb have not yet been established. Recently, alternative functions of (d) O_2 _sensing and (e) nitric oxide scavenging were proposed [14]. Another recent paper [15] seemed to have totally scrambled the past long-term disputes about the physiological significance of Mb. It was shown using gene-knockout technology that mice without Mb are fertile, exhibit normal exercise capacity, and have a normal ventilatory response to low O_2 _levels, suggesting that Mb is not essential for apparently normal cardiovascular and musculoskeletal function in a terrestrial, homoiothermic mammal. However, it has been reported that the disruption of Mb results in the activation of multiple compensatory mechanisms such as increases in Hb concentration, hematocrit, coronary flow, coronary reserve, and capillary density [16]. Further, a Mb-like hemoprotein, neuroglobin, has been found in the vertebrate brain [17] contrary to the long-held belief that Mb is restricted to vertebrate cardiomyocytes and oxidative skeletal myofibers. These studies imply that further investigations are required to reveal the physiological role of Mb in intact organs.

In contrast to Mb, which shows a rectangular hyperbolic ODC, the vertebrate Hb, a tetramer carrying four O_2 _binding sites, shows a sigmoid ODC that is described in terms of a four-step cooperative O_2 _binding. It is widely accepted that the sigmoid ODC enables Hb to transport O_2 _with high efficiency: it is nearly fully saturated with O_2 _in the lungs and it unloads O_2 _sensitively depending on decreases in the partial pressure of oxygen (PO_2_) in peripheral tissues. Here, no convincing explanation has been given for the question: does the hyperbolic ODC of Mb have any physiological adequacy or reasonability? The Bohr effect of Hb (pH dependence of O_2 _affinity) has physiological significance, in that it enhances O_2 _unloading from Hb in the capillaries where pH tends to decrease and in that it increases the solubility of CO_2 _as bicarbonate in the venous blood through deoxygenation-induced uptake of protons by Hb. In contrast, Mb lacks the Bohr effect and it had long been believed that Mb was a totally non-allosteric protein, although recently lactate, a metabolic product, was found to cause a right-shift of the ODC for horse and sperm whale Mbs [18].

**Figure 1:**
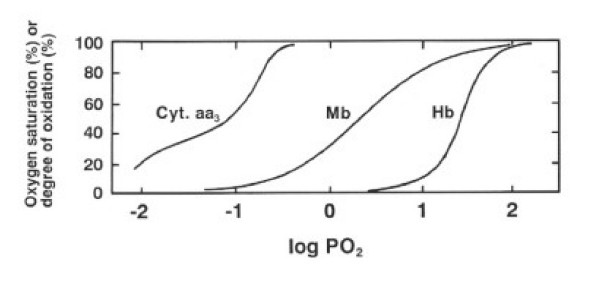
Oxygen dissociation curves (ODCs) for Hb (whole blood) and Mb and oxidation curve for Cyt. aa_3 _(at 37°C). PO_2_, partial pressure of oxygen in mmHg. Data from Imai [36].

It is well established that the O_2 _affinity of Mb is higher than that of Hb but lower than that of Cyt. aa_3_, as known from the relative positions of the ODCs for Mb and Hb and the oxidation curve for Cyt. aa_3 _(Fig. 1). This fact led one to the idea that Mb acts as an intracellular O_2 _transfer agent from Hb (vascular space) to Cyt. aa3 (mitochondria). Here, one must not overlook an important fact. The three curves in Fig. 1 are drawn with the same PO_2 _scale. Therefore, they give O_2 _saturation (Y) or the degree of oxidation for the individual proteins when dissolved in the same solution and are in equilibrium with oxygen at the given PO_2 _value. However, *in vivo*, they sense different PO_2 _values due to the presence of a PO_2 _gradient along the path from the inside of red cells to the mitochondria in myocytes. Thus, the relative positions of the three curves in Fig. 1 must be considered with this precaution, and direct *in vivo *observations of Y or the degree of oxidation for these three individual proteins are required to get insight into their ensemble functional roles. Recently, using ^1^H nuclear magnetic resonance spectroscopy, Mole et al. [19] and Richardson et al. [20] directly observed Y for Mb in human skeletal muscles under exercise of different intensities or during breathing of air with different O_2 _contents. In these studies, Mb was used as an indicator of intracellular PO_2_, and no attention was paid to the relation between Mb saturation and capillary PO_2_.

In the present study, we directly measured Y for Mb in isolated rat hindlimb muscles, perfused with a Hb-free medium, under vigorous changes in respiration conditions. We plotted the Y values as a function of buffer PO_2 _and found that the apparent ODC thus plotted for skeletal muscle Mb was rectangular hyperbolic under a suppressed metabolic activity condition but it became sigmoid under enhanced metabolic activity conditions, realizing virtually cooperative O_2 _binding by monomeric Mb.

## Methods

### Muscle perfusion

All experimental procedures were performed according to the institutional guidelines for animal care and use of the Committee for Animal Care of Osaka University and the Japanese Physiological Society. Male Wistar rats (250 to 300 g body weight, N = 12) fed on a commercial diet were used. Rats were anesthetized with sodium pentobarbital (30 mg/kg body wt., intraperitoneal injection). Preparation of the isolated rat hindlimb and the perfusion apparatus were described previously [21,22]. Surgery was modified from those of Ruderman et al. [23] and Shiota et al. [24]. After a midline abdominal incision, the superficial epigastric vessels were ligated. The abdominal wall was then incised from the pubic symphysis to the xiphoid process. The spermary, testis, and inferior mesenteric arteries and veins were ligated, and the spermaries, the testises, and part of the descending colon were excised, together with contiguous adipose tissue. The caudal artery and internal iliac artery and vein were also ligated. Ligature were placed around the neck of the bladder, the coagulating gland and the prostate gland. While carefully removing the skin covering the lower half of the animal, the vessels that supply the subcutaneous region were ligated. Then, the inferior epigastric, iliolumbar and renal arteries and veins were ligated as well as the coeliac axis and portal vein. Further, a ligature was also placed around the tail. A hemoglobin-free Krebs-bicarbonate buffer (NaCl, 115 mM; KCl, 5.9 mM; MgCl_2_, 1.2 mM; NaH_2_PO_4_, 1.2 mM; Na_2_SO_4_, 1.2 mM; NaHCO_3_, 25 mM; CaCl_2_, 2.5 mM; glucose, 10 mM; pH 7.4) containing 4% (w/v) polyvinylpyrrolidone (PVP-40T; average M.W., 40,000; Sigma) was perfused from the abdominal aorta in the flow-through mode at a constant flow rate of 1.0 ml/min/g muscle. Perfusate and muscle temperature were maintained at 25 ± 0.5°C. The effluent was collected from the inferior vena cava in order to measure the O_2 _uptake rate. PO_2 _in the influent and the effluent buffers was monitored with an oxygen electrode. The rate of O_2 _uptake was calculated from the flow rate and the difference in O_2 _concentration between the influent and the effluent buffers. Before each measurement, the rat hindlimb was perfused with the buffer equilibrated with 95% O_2 _+ 5% CO_2 _for 30 min. Then, the O_2 _concentration in the perfusate was decreased stepwise by mixing a buffer equilibrated with 95% O_2 _+ 5% CO_2 _and another equilibrated with 95% N_2 _+ 5% CO_2_, and the measurement was started. As required, potassium cyanide or 2,4-dinitrophenol was infused to modify the O_2 _uptake rate of the perfused muscle. During each measurement of about 60 min, the perfusion pressure remained nearly constant at 73–78 mmHg. All chemicals used were of analytical reagent grade.

### Spectrophotometric measurement of myoglobin oxygenation

A computer-controlled rapid scanning spectrophotometer (USP-501, Unisoku, Osaka, Japan) was used to measure the oxygenation of Mb in the transmission mode [21,22]. The light beam was focused on the thigh (quadriceps) through a 5-mm-diameter light guide. The transmitted light was conducted to the spectrophotometer through another 5-mm-diameter light guide. Visible difference spectra in the range of 500–650 nm were recorded when O_2 _uptake in the hindlimb muscle reached a constant value after every stepwise change in the O_2 _concentration of the buffer.

### Analysis of data

Changes in the O_2 _uptake rate were analyzed using a rectangular hyperbolic curve equation: V = V_max_(PO_2_/P_V50_)/{1 + (PO_2_/P_V50_)}. Here, the maximal rate of O_2 _uptake (V_max_) and effluent buffer PO_2 _at half maximal O_2 _uptake (P_V50_) were obtained from the slope (1/V_max_) and the ordinate intercept (P_V50_/V_max_) of the Hanes-Woolf plot (effluent PO_2_/V vs. effluent PO_2_). Changes in oxygen saturation of Mb (Y) were analyzed using the Hill equation [25], Y = PO_2_^n^/(PO_2_^n ^+ P_Y50_^n^), where P_Y50 _is PO_2 _at half saturation of Mb (Y_50_) and n is the Hill coefficient. In the original Hill equation, n was treated as a constant. This equation expressed the ODC of Mb well but not the ODC of Hb because the Hill plot for Hb deviated from a straight line at both extremes. To make the Hill plot applicable to Hb, Wyman [26] extended the equation by linearizing it in the form: log {Y/(1 - Y)} = n (log PO_2 _- log P_Y50_) where n was treated as a variable. This extension allowed cooperativity measured by n to vary depending on Y.

## Results

### Oxygen uptake by perfused muscle in different respiration states

**Figure 2:**
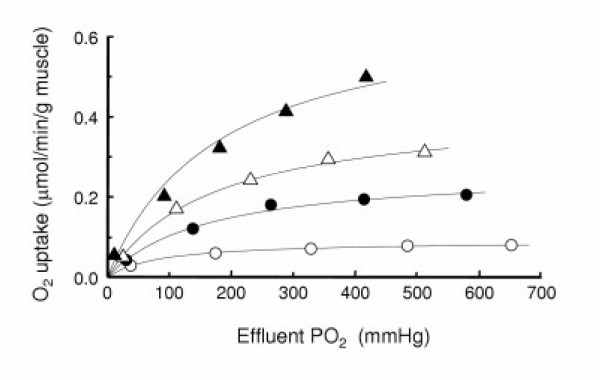
Steady-state O_2 _uptake rate (V) of perfused rat hindlimb muscles as a function of effluent buffer PO_2_. The rat hindlimb was perfused with Krebs-bicarbonate buffers containing no additive (●) as control, 0.4 mM of KCN (○) for suppression of muscle respiration, and 5 μM (△) or 10 μM (▲) 2,4-dinitrophenol for stimulation of muscle respiration. Symbols express observed points. Each plot is the mean of experiments using three animals, and the errors for each data point are less than the size of symbols. The solid lines were calculated using a rectangular hyperbolic curve: V = V_max_(PO_2_/P_V50_)/{1 + (PO_2_/P_V50_)}. The values of P_V50 _and V_max _are given in Table 1 which also includes the maximal values of influent and effluent PO_2_.

Figure 2 shows the steady-state O_2 _uptake rate (V) of a perfused muscle. The respiration rate of the muscle was varied by controlling mitochondrial respiration activity by about 7.5-fold (compare the V_max _values described below) from a suppressed state with an inhibitor (KCN) of mitochondrial respiration to enhanced states with two levels of an uncoupler (2,4-dinitrophenol) of mitochondrial respiration. Three preparations of muscle were used for the experiments in each mitochondrial activity state. The actual PO_2 _values of the influent and effluent buffers at the maximal O_2 _inflow rate are listed in Table 1. Changes in the value of V were well expressed by a rectangular hyperbolic curve as a function of effluent buffer PO_2 _(Fig. 2). Table 1 also gives the estimated V_max _and P_V50 _obtained from these data as described in Materials and Methods. V_max _and P_V50 _became larger by approximately 7.5-fold and 2-fold, respectively, for the maximal increase in respiration activity. With elevation of respiration activity, the critical PO_2_, at which O_2 _uptake of perfused hindlimb muscle starts to decrease, increased to higher values. This indicates that, under higher respiration activity, O_2 _supply to the perfused muscle was limited even at very high influent PO_2 _(~700 mmHg). This situation occurred because the flow rate of the perfusate and the capillary PO_2 _were controlled independently of the respiration activity state so that the PO_2 _gradient between the perfusate and the mitochondria became larger at higher respiration states.

**Table 1:** Values of muscle perfusion parameters and Mb oxygenation parameters in various tissue respiration states

Respiration state:	Suppressed (0.4 mM KCN)	Control	Enhanced (5 μM DNP^a^)	Enhanced (10 μM DNP)
Influent PO_2_	700	700	700	700
Effluent PO_2_	652 ± 14	579 ± 6	512 ± 15	418 ± 18
V_max_^b^	0.09	0.27	0.42	0.68
P_V50_^c^	83	160	170	180

a, 2,4-dinitorophenol; b, Maximal value of steady-state O_2 _uptake rate (V) at infinite influent PO_2 _(in μmol/min/g muscle); c, effluent PO_2 _at V = half V_max _(in mmHg). Values of V_max _and P_V50 _were obtained from solid lines shown in Figure 2.

### Relationship between effluent buffer PO_2 _and Mb oxygenation in perfused muscle

**Figure 3:**
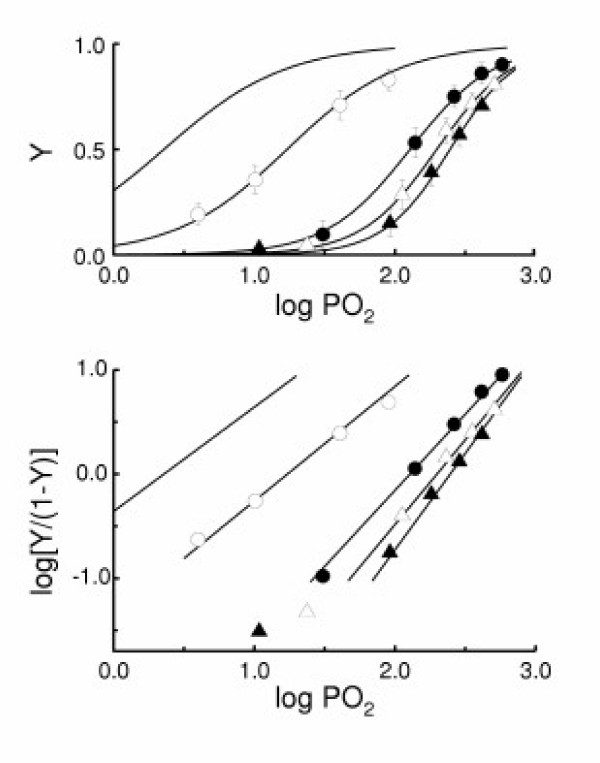
Apparent ODCs for Mb in perfused muscle at various steady-state O_2 _uptake levels. PO_2 _is the same as in Fig. 2. **A**, ODCs as O_2 _saturation (Y) plotted against log PO_2_. The lines were calculated from the Hill equation (see below). Each ODC was obtained from three animals (three muscle preparations). Symbols (mean ± SD) express observed points and their meaning is the same as in Fig. 2. The lines without symbols are the ODCs for Mb in non-respiring muscle [21]. **B**, Apparent ODCs as expressed by the Hill plot which is based on the linearized Hill equation [25]: log {Y/(1 - Y)} = n (log PO_2 _- log P_Y50_). The slope of the plot (n) was constant and expressed as n_app _in the present paper. The n_app _and intercept values obtained from the Hill plots are listed in Table 2.

Figure 3A shows ODCs for Mb in the perfused muscle. Here, Y is plotted against effluent buffer PO_2_. These ODCs are apparent in the sense that the PO_2 _is not the value at the location where Mb is working. The curve was shifted to the right and became steeper as muscle respiration activity was enhanced. These oxygenation data were further expressed by means of the Hill plot (log [Y/(1-Y)] vs. log PO_2_), yielding linear plots (Fig. 3B). The effluent buffer PO_2 _at half saturation (P_Y50_) and the slope of the Hill plot (the Hill coefficient, n) obtained from these plots are listed in Table 2, where n is expressed as n_app _(apparent n). The P_Y50 _value became larger with an increase in muscle respiration activity. The log P_Y50 _value was nearly linearly related to the log P_V50 _value (not shown). The n_app _value also increased from 1.10 in the suppressed respiration activity state to 1.85 in the 7.5-fold enhanced respiration activity state.

**Table 2:** Linear regression parameters for Hill's plot of myoglobin oxygenation in perfused rat hindlimb muscle, log [Y/(1-Y)] = k + n_app._* log [effluent PO_2_]

Respiration state:	Suppressed	Control	Enhanced	Enhanced
	(0.4 mM KCN)		(5 μM DNP^a^)	(10 μM DNP)
Slope, n_app._*	1.10 ± 0.10	1.46 ± 0.06	1.63 ± 0.15	1.85 ± 0.05
Intercept, k*	-1.36 ± 0.13	-3.08 ± 0.13	-3.75 ± 0.34	-4.43 ± 0.12

* 95% confidence level.

**Figure 4:**
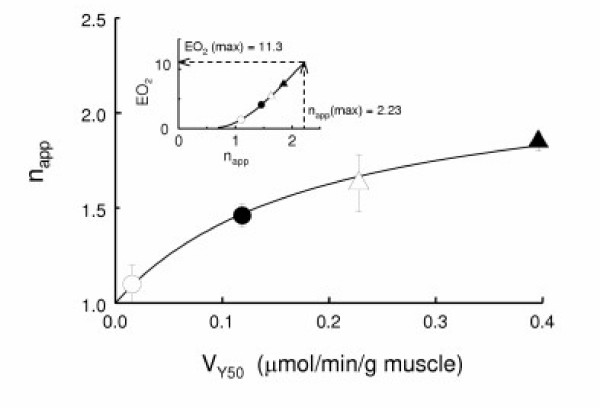
Relationship between n_app _and steady-state O_2 _uptake rate at Y = 50% (V_Y50_). The V_Y50 _values were obtained from the hyperbolic curves in Fig. 2. The symbols (mean ± SD) are as in Fig. 2. The solid line, which was calculated from the equation: n_app _= 1 + 1.23*V_Y50_/(0.193 + V_Y50_), simulates the observed dependence. The maximal value of n_app _at infinite V_Y50 _is 2.23, which was obtained using the Hanes-Woolf plot, V_Y50_/(n_app _- 1) vs. V_Y50_. The inset figure shows the relationship between n_app _and O_2 _transport efficiency (EO_2_) (see Text).

**Figure 5:**
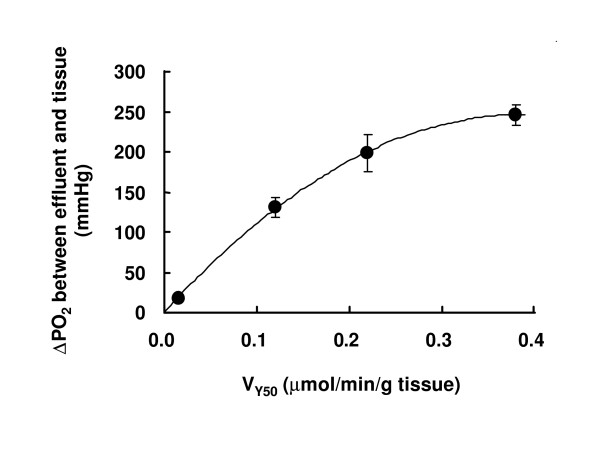
Correlation between O_2 _consumption at 50% of Mb oxygenation (V_Y50_) and calculated O_2 _gradient from vascular to cytoplasmic space in perfused hindlimb muscle (ΔPO_2_). To calculate ΔPO_2_, PO_2 _= 2.3 mmHg at Y = 50% of Mb in the perfused muscle was used [21]. ΔPO_2 _= -1635* V_Y50 _+ 1269 (r^2 ^= 0.9995).

Since this virtual cooperativity is of particular interest, its relation to O_2 _uptake rate was further examined. Figure 4 shows the dependence of n_app _on V at the half O_2 _saturation point of Mb (V_Y50_). The n_app _value asymptotically increased from unity for the non-respiring state to 2.23 at infinite V_Y50_. These results indicate that the apparent ODC of Mb in the perfused muscle is transformed from a hyperbolic curve to a sigmoid curve depending on the magnitude of tissue respiration. Effect of the Hill coefficient (n) on ratio of substrate (or ligand) concentrations necessary to change enzyme activity from 90% to 10% of maximal can be expressed with a parameter, R (= 81^1/n^) [27]. Here, the O_2 _transport efficiency (EO_2_) was estimated as ratio of the parameter at n_app _= 1 to that at a given value of n_app _(Fig. 4 inset). Figure 5 shows the effect of muscle respiration on the O_2 _gradient between effluent and the perfused tissue. Assuming the effluent buffer PO_2 _approximates the capillary PO_2_, the calculated O_2 _gradient from capillary to cytoplasmic space (ΔPO_2_) is plotted against V_Y50_. Here, the P_50 _value of Mb in the perfused muscle was 2.3 mmHg [21]. ΔPO_2 _increased with the increase in V_Y50_. This result indicates the presence of a large O_2 _diffusion barrier between capillary lumen and cytoplasmic space.

## Discussion

In the present study, by using computer-controlled rapid scanning fiber-optic spectrophotometry, I directly measured Y for Mb in isolated rat hindlimb muscles under extensive changes in respiration rate caused by mitochondrial activity control or perfusate PO_2 _control. It is assumed that capillary PO_2 _may be approximated by effluent PO_2 _in the present experiment, and I plotted the Y values as a function of effluent buffer PO_2_. Thereby, I expected that this treatment enabled a meaningful comparison of the ODCs for Mb and Hb. I found that thus plotted apparent ODC for skeletal muscle Mb was hyperbolic under a suppressed metabolic activity condition whereas it became sigmoid under enhanced metabolic activity conditions, realizing virtually cooperative oxygenation of the monomeric Mb.

It is generally accepted that cooperative O_2 _binding by Hb is advantageous for efficient O_2 _transfer from the alveolar gas to red cells and from red cells to peripheral tissues. Based on the Hill equation, Graby and Meldon [28] showed that an *n *value (here, *n *is a constant) of 1.5 to 2.0 is more favourable for minimizing the change in blood flow under resting conditions than the normal *n *value of 2.5 to 3.0, whereas an *n *value as large as 3 is beneficial for a large amount of O_2 _extraction under vigorous exercise. Kobayashi et al. [29] showed that, under resting conditions, O_2 _release from Hb becomes most sensitive to PO_2 _change at Y = 38% where cooperativity measured by *n *(here, *n *is a variable of PO_2_) is not maximal, whereas it becomes less sensitive at the mixed venous blood PO_2 _where Y is around 70% and cooperativity is nearly maximal. These reports indicate that, under resting conditions, the blood reserves an O_2 _transport capacity to meet possible increases in O_2 _demand, e.g. under exercise conditions, and the sigmoid character of ODC becomes more important under such conditions. This situation is realized by maintaining Y at a rather high level (70%) below which the Y value drops sharply upon PO_2 _decrease within the very steep middle portion of ODC.

The present study has clearly shown that the apparent ODC for Mb in intact skeletal muscle is sigmoid, the n_app _value being 1.46 under the control condition (Table 2) and 2.23 under the maximal respiratory condition (Fig. 4). These n_app _values greater than unity imply that the muscle Mb binds O_2 _in a virtually cooperative manner with variation of effluent buffer PO_2_. This phenomenon implies that the sensitivity of Y for Mb to vessel PO_2 _change becomes higher for increased O_2 _demands than for normal O_2 _demand. In addition to this effect, the rightward shift of the ODC upon increases in oxygen demand will undoubtedly enhance O_2 _unloading from Mb. These effects will facilitate Mb-mediated O_2 _transfer from Hb to Cyt. aa_3_, especially for heavy O_2 _demands. Based on the Hill equation, the O_2 _transport efficiency of Mb in the perfused muscle is estimated to increase ca. 4-fold under the control condition and ca. 11-fold under maximally respiring condition (Fig. 4 inset).

The Mbs isolated from body wall or radular muscle of a limited number of annelidan and molluscan species are dimers and show some cooperativity in oxygen binding (1 < n < 2) but no Bohr effect [30]. The physiological significance of these dimeric Mbs is unknown. As shown in the present study, the ODC of monomeric Mb can exhibit virtual cooperativity and O_2 _demand-dependent shifts. The virtually cooperativity and O_2 _demand-dependent shifts of Mb oxygenation *in vivo *are probably common features at least for vertebrate Mbs, and this may provide a basis for explaining why the vertebrate Mb has been preserved as a monomer during molecular evolution.

The virtual cooperativity in Mb oxygenation observed in the present study is explained in terms of the PO_2 _gradient along the O_2 _diffusion path. If the tissue O_2 _demand was null, then the PO_2 _gradient would be absent and the apparent ODC for Mb would be identical with the real ODC for Mb in solution. At a steady state with a certain level of O_2 _demand a PO_2 _gradient develops across red cell membrane, blood plasma, capillary wall, sarcolemma and sarcoplasm, making the PO_2 _sensed by Mb lower than the capillary PO_2_. Then, the apparent ODC will be shifted toward the right because a capillary PO_2 _value higher than the PO_2 _value sensed by Mb is needed to maintain the same Y value as that which occurs in the absence of a PO_2 _gradient. When the tissue O_2 _demand is kept constant, the ratio of capillary PO_2 _to sarcoplasm PO_2 _will become larger at low capillary PO_2 _than at high capillary PO_2_. This will cause a more extensive rightward shift of the apparent ODC in the low saturation range than in the high saturation range, making the curve steeper than the real one. Increase in tissue oxygen demand will enhance this mechanism and make the curve more right-shifted and sigmoid. All the apparent ODCs observed in the present study are shifted toward the right compared to the real one measured for Mb in solution (Fig. 3).

At present, detailed explanations for this cooperative mechanism is difficult. However, it could be argued that heterogeneous oxygenation in tissue [31] and in single myocytes [32] might be responsible in part for the shift and the shape change of the Mb ODC, and might also enhance intercellular O_2 _transfer, i.e., re-distribution of O_2 _among adjacent myocytes, although we adopted high and constant flow rate perfusion conditions (i.e., about 50 times higher than normal blood flow) and, thus, the perfused vessels of muscle were always passively dilated.

Unfortunately, it is not practical to use a Hb solution or a red cell suspension as the perfusate in our experiments because the absorption spectra for Hb and Mb are too similar and independent observations of Mb oxygenation are not feasible, especially when the concentration of Hb is much higher than that of Mb. To overcome the problem that the O_2 _solubility of the buffer is much smaller than that of a Hb solution or a red cell suspension two strategies were employed: one was to make the PO_2 _of the influent buffer as high as that of water vapor-saturated O_2 _(ca. 700 mmHg) and the other was to use a high flow rate for the buffer, which was about 50 times higher than that of normal blood flow. As a result, the inflow of O_2 _was about 5 times larger than that of the tissue O_2 _consumption at the control metabolic rate. The large O_2 _diffusion barrier (see Fig. 5) and the high PO_2 _of the influent buffer (and consequently, the high capillary PO_2_) are an additional (and probably, major) cause for the rightward shift of the apparent ODC of Mb. The apparent ODCs of Mb in the control and enhanced respiration activity states (Fig. 3A) are right-shifted compared to the whole blood ODC (Fig. 1). One may suppose that Mb cannot work when the capillary PO_2 _is in the physiological range (40 to100 mmHg) because its O_2 _saturation is too low to function, as judged from Fig. 3A. However, the actual apparent ODCs for Mb in muscles with blood circulation will be shifted much more toward the left compared to those shown in Fig. 3A, and Mb can be saturated with O_2 _to practical levels. The important point is that the difference in *in vivo *O_2 _saturation between Hb and Mb is not so large as that expected from the ODCs in Fig. 1. In fact, Y for Mb in working muscles is less than around 50% [19,20,31-33]. Red blood cell (RBC) in perfusion buffer appears to exert considerable effects on intracellular oxygenation in the beating heart [34], probably due to the facilitated O_2 _transfer by RBC motion within capillary lumen [35]. Therefore, the virtually cooperative oxygenation of Mb might be only demonstrated in organs perfused with RBC-free medium. However, it is well known that the blood flow in the capillary bed is not constant and frequently only plasma flow is observed. In this case, the virtually cooperative oxygenation of Mb may play a significant role for O_2 _transfer from capillary to mitochondria.

In summary, I found that the ODC for Mb in intact skeletal muscle is sigmoid and right-shifted. This virtually cooperative O_2 _binding by Mb and the right-shift of ODC become more marked as tissue respiration activity is increased. Hence, increase in O_2 _demand in tissues makes the O_2 _saturation of Mb more sensitive to capillary PO_2 _change and enhances Mb-mediated O_2 _transfer from red cell to motochondria. The virtual cooperativity and O_2 _demand-dependent shifts of ODC may give a basis for explaining why Mb has been preserved as a monomer during molecular evolution. Preservation of a monomeric structure may be required to retain multi-functional role of Mb *in vivo*.
